# Primer on tumor immunology and cancer immunotherapy

**DOI:** 10.1186/2051-1426-1-12

**Published:** 2013-07-29

**Authors:** Timothy J Harris, Charles G Drake

**Affiliations:** 1 Department of Radiation Oncology & Molecular Radiation Sciences, Johns Hopkins University School of Medicine and Sidney Kimmel Comprehensive Cancer Center, Baltimore, MD, USA; 2 Department of Oncology and Brady Urological Institute, Johns Hopkins University School of Medicine and Sidney Kimmel Comprehensive Cancer Center, 1650 Orleans St., CRB I #410, Baltimore, MD 21231, USA

**Keywords:** Immunotherapy, Cancer vaccine, Immune checkpoint, Adoptive T cell therapy

## Abstract

Individualized cancer therapy is a central goal of cancer biologists. Immunotherapy is a rational means to this end—because the immune system can recognize a virtually limitless number of antigens secondary to the biology of genetic recombination in B and T lymphocytes. The immune system is exquisitely structured to distinguish self from non-self, as demonstrated by anti-microbial immune responses. Moreover the immune system has the potential to recognize self from “altered-self”, which is the case for cancer. However, the immune system has mechanisms in place to inhibit self-reactive responses, many of which are usurped by evolving tumors. Understanding the interaction of cancer with the immune system provides insights into mechanisms that can be exploited to disinhibit anti-tumor immune responses. Here, we summarize the 2012 SITC Primer, reviewing past, present, and emerging immunotherapeutic approaches for the treatment of cancer—including targeting innate versus adaptive immune components; targeting and/or utilizing dendritic cells and T cells; the role of the tumor microenvironment; and immune checkpoint blockade.

## Introduction

The immune system is able to distinguish self from non-self, and is able to vigorously attack non-self and infected self tissues. This is the basis for anti-microbial responses. The immuno-editing theory suggests that the immune system is able to recognize and eradicate subclinical tumors, but at some point equilibrium is reached and the tumor remains in situ, in a state of balance with a partially efficacious response [1]. Unfortunately, many tumors escape from this equilibrium state, and cancer becomes clinically apparent. The goal of the cancer immunotherapist is to understand the mechanisms by which cancer is able to escape the immune system and to therapeutically intervene at critical points to promote anti-tumor immune responses. Broadly, such interventions fall under the umbrella of “immunotherapy” and can include cancer vaccines, cytokine therapy, the administration of monoclonal antibodies to block immune checkpoints, and others. The Society for Immunotherapy of Cancer (SITC) organized a Primer on Tumor Immunology and Cancer Immunotherapy with the assistance of Willem W. Overwijk, PhD (Innate Immunity and Inflammation), Madhav Dhodapkar, MD (Dendritic Cells), Helen Chen, MD (Immunology of Antibodies as Therapy), Susan M. Kaech, PhD (Effector and Memory T Cell Differentiation), Thomas F. Gajewski, MD, PhD (Immunobiology of the Tumor Microenvironment), Jonathan Powell, MD, PhD (T Cell Intracellular Signaling), Pedro J. Romero, MD (Tumor Antigen and Immunogenicity), Charles G. Drake, MD, PhD (Coinhibition and Costimulation), and Cassian Yee, MD (Adoptive Cellular Therapy). Mario Sznol, MD and Charles Drake, MD, PhD served as co-organizers.

## Review

### Innate immunity and inflammation

The innate immune system recognizes pathogens based on repeated patterns and responds quickly with a variety of effector mechanisms. This is in contrast to the adaptive immune system, consisting of T and B cells, which responds more slowly, but which is more specific. An innate immune response is often evidenced by Inflammation: a local response to tissue injury—defined by the presence of Rubor (redness), Calor (heat), Dolor (pain), and Tumor (swelling). The innate immune response functions to eradicate invasive pathogen; limit the spread of infection; initiate adaptive immune responses involving T and B cells; and to initiate tissue repair. Immune responses and inflammation are generally advantageous for the host, and may include suppressing growth of smaller tumors. Interestingly however, inflammation can also promote neoplastic transformation and tumor progression. For example, in a genetically engineered lung cancer model using mice with a mutation in K-ras, cigarette smoke induced inflammation and tumor development through the activation of myeloid cells [2]. In a preclinical model of squamous cell carcinoma related to HPV E6/E7, chronic inflammation caused by lymphocytes and Fc Gamma Receptor signaling on myeloid cells was responsible for malignant transformation, and tumorigenesis could be abrogated via lymphocyte depletion or Fc Gamma Receptor blockade [3]. Mutations within tumors can play a role in this process; BRAF mutations drive tumor cells to produce pro-inflammatory cytokines like VEGF, IL-10, and IL-6, while at the same time decreasing expression of anti-tumor cytokines such as IL-12 [4]. Mutated BRAF also promotes the secretion of IL-1α and IL-1β, innate inflammatory mediators which can drive tumor cells to protect themselves from immune attack by up-regulating molecules that inhibit the function of anti-tumor lymphocytes [5]. Another molecular mechanism linking chronic inflammation to cancer progression involves a transcription factor known as STAT3 (signal transducer and activator of transcription 3). In areas of chronic inflammation, tumors up-regulate activated (phosphorylated) STAT3, which, in addition to being anti-apoptotic, drives expression of cytokines that dysregulate anti-tumor immune responses [6]. Tumors can also cause systemic immunosuppression as noted in preclinical models demonstrating an increase in splenic myeloid suppressor cells, which are a specialized population of innate myeloid cells [7,8]. Taken together, these data provide examples of how the innate immune system can promote tumor progression, and suggest pathways for intervention.

### Dendritic cells

Dendritic cells (DC) link the innate immune system to the adaptive immune response. These cells dwell in the tissues, continually sampling the microenvironment and taking up antigens primarily through pinocytosis. When the innate immune system is activated in their vicinity, DCs sense this as “danger” [9], cease antigen uptake and travel to local lymph nodes, where their role is to present antigen to specific T lymphocytes. The microenvironment in which a DC acquires antigen determines whether the DC will have the capacity to activate an antigen-specific lymphocyte or to tolerize the lymphocyte. In addition to pinocytosis, immature DCs are also able to internalize antigen through Fc receptor-mediated endocytosis, a process in which Fc receptors on the DC bind antibody-bound antigen. Emerging data suggest that the subtype of Fc receptor involved in antigen internalization helps to determine whether the response to that antigen will be activating or inhibitory [10]. As introduced above, the ultimate outcome of the lymphocyte-DC interaction is based primarily on the state of the DC. DCs can produce distinct cytokine groups that can skew T lymphocytes toward divergent functions. Moreover, triggering distinct Toll-like receptors (TLRs) on DCs elicits different cytokine profiles and different immune responses. Signaling through TLRs 4, 5, or 11 results in DC production of IL-12, which in turn skews T-cells towards a T_H_1 phenotype capable of promoting anti-tumor immune responses [11]. Signaling through TLRs 1, 2, or 6 causes DCs to produce IL-10, which in turn promotes T-cell development towards regulatory or T_H_2 phenotypes incapable of promoting anti-tumor immune responses [11]. In mice, a subset of DCs that express CD8 is primarily responsible for priming anti-tumor immune responses [12]. Their human equivalent is thought to be CD141^+^ DCs [13-16]. These subsets are known to produce IL-12 and cross-present antigens to lymphocytes.

How can our knowledge of DC biology be used to develop immunotherapy for patients? While it is clear that activation of these cells is desirable, there are two general approaches to achieve that end: *ex vivo* and *in vivo* activation. *Ex vivo* strategies for DC-based immunotherapies include generation of DCs from circulating monocytes via subsequent culture, as well as procedures in which DCs are derived from circulating CD34^+^ hematopoetic stem cells (HSCs). In the U.S., an immunotherapy based on *ex vivo* activated DCs has been FDA-approved to treat patients with metastatic prostate cancer. This product, sipuleucel-T is generated by incubating a patient’s monocytes with a fusion protein that links the target antigen (Prostatic Acid Phosphatase) to the cytokine GM-CSF; here GM-CSF serves to mature the monocytes toward DCs, and assists in internalization of the antigen. After *ex vivo* incubation, the mixed cellular product, including maturing DCs, is re-infused into patients. In a randomized control trial for prostate cancer patients, this product resulted in a 4.1 month improvement in median survival compared to placebo (HR 0.78; P = 0.03) [17]. Another common strategy for DC-based immunotherapy involves maturation of immature monocytes into DCs by culturing them for several days in the presence of GM-CSF and IL-4. The DCs are then loaded with tumor-specific peptides or in some variation with whole protein antigens which they must subsequently process and present [18]. While *ex vivo* stimulation of DCs often results in quantifiable immune and clinical responses with no dose limiting toxicities, the overall clinical response rates to this therapeutic approach have remained somewhat low [19]. The other major strategy under study is to target antigens specifically to DCs *in vivo*. This routinely involves the use an adjuvant (e.g. TLR agonist) in combination with signaling antibodies (e.g. anti-CD40, anti-DC-SIGN, anti-MMR, anti-DEC-205) and tumor-specific antigen [20]. In summary, an evolving understanding of DC biology has led to the first commercially approved cellular immunotherapy for the treatment (not prevention) of a solid tumor, and further developments in this field are likely.

### Antibodies as therapy

Monoclonal antibodies are now widely utilized in the treatment of a number of tumor types; pertinent examples including trastuzumab (anti-Her-2) for the treatment of breast cancer, rituximab (anti-CD20) for the treatment of lymphoma, and the recently approved immunoconjugate T-DM1, which fuses trastuzumab to a highly potent chemotherapy, emtansine (DM1 [deacetyl maytansine]) to facilitate local delivery and minimize systemic toxicity [21]. Antibody-based immunotherapeutics can be exquisitely specific treatment tools, based on the diverse and nanomolar level affinity of the Fv region of the antibody for its target, as well as the ability of the Fc region to engage components of the host immune system. How do monoclonal antibodies work? The mechanisms of action of unconjugated monoclonal antibodies include blocking a pro-survival signal, as well as facilitating tumor cell destruction by the binding of the Fc portion of the antibody to Fc Receptors on natural killer (NK) cells—promoting the ability of NK cells to lyse their targets through a process known as antigen-dependent cytotoxicity (ADCC). Monoclonal antibodies can also mediate cytotoxicity by binding to complement receptors on effector cells, a process known as complement-dependent cytotoxicity (CDCC). The Fc portion of a monoclonal antibody plays a major role in determining the immune mechanisms induced, with monoclonal antibodies of the human IgG4 isotype primarily functioning as “blockers”. One interesting aspect involved in the development of monoclonal antibodies for the clinic involves their affinity, while higher antibody affinity results in increased target engagement and ADCC, higher affinities can also result in decreased tumor penetration and compromised efficacy [22-24].

Several recent clinical developments highlight the increasingly prominent role of antibody-based therapy in cancer. In an important recent result, Yu et al. showed an 11% absolute benefit in 2-year survival in patients with advanced neuroblastoma treated with a combination of IL-2, GM-CSF, and an antibody targeting GD2 (disialoganglioside 2) (P = 0.02) [25]. As discussed above, a great deal of recent interest involves conjugating monoclonal antibodies to either a cytotoxic agent, examples include brentuximab vedotin (anti-CD30-MMAE [monomethyl auristatin E]) for anaplastic large cell and Hodgkin lymphoma, trastuzumab emtansine (anti-HER2-DM1) for breast cancer, and glembatumumab vedotin (anti-GPNMB-MMAE) for breast cancer [21,26,27]. In addition, T cells can be re-engineered to express chimeric (antibody-based) antigen receptors (CARs) to target the powerful killing machinery of cytotoxic lymphocytes directly to tumor antigen [28]. CAR transformed T cells have been developed against a variety of antigens, including CEA, CAIX, EGFR, HER2, CD19, and CD20, but serious adverse events have been reported [29,30]. In a particularly relevant report, Porter et al. recently showed that a CAR specific for CD19 could mediate a major clinical response in a patient with chronic lymphocytic leukemia [29]. Another fascinating application of monoclonal antibody technology involves the engineering of bi-specific antibodies, in which one arm carries specificity for a tumor antigen, and the other arm is specific for the CD3 complex on T cells. The idea behind this technology is to physically co-localize lymphocytes to tumors, inducing anti-tumor T cell responses. Bi-specific antibodies against CD19 (blinatumomab) have shown promise in Phase I-II studies [31,32].

### Tumor microenvironment

*In vivo*, a tumor is significantly more complex than a simple group of clonogenic cells. The three-dimensional mass that is appreciated on imaging studies contains, in addition to tumor cells, extracelluar matrix components, supportive stromal cells (e.g. neovasculature, fibroblasts, and macrophages), and a number of inflammatory cells. In terms of mounting an anti-tumor immune response, there is further complexity involved, since the priming and effector phases of the immune response are separated by time and space. While priming occurs in lymph nodes, the effector functions must operate within the tumor mass. Potential barriers to anti-tumor responses encountered during the priming phase include a paucity of “danger” signals from innate immune cells, poor recruitment of DCs for cross-presentation, and inadequate expression of costimulatory ligands on tumor cells or APCs. Potential barriers to efficacy during the effector phase involve inadequate recruitment of activated effector T cells secondary to abnormal vascular endothelial cells and/or chemokines, the presence of dominant immune inhibitory mechanisms capable of abrogating T cell effector function (e.g. the inhibitory receptors PD-1 and CTLA-4), extrinsic suppressive cells (T_REGs_, myeloid-derived suppressor cells), metabolic inhibitors (IDO, arginase), and inhibitory cytokines (IL-10, TGF-β) [33].

The genetic profile of the tumor microenvironment and its potential correlation with anti-tumor immune responses has become an area of increased study in recent years. In one preclinical study of metastatic melanoma, the expression of a subset of chemokines were associated with CD8^+^ T cell infiltration [34]. In patients with metastatic melanoma, the expression of T cell markers and chemokines correlated with response to a DC-based vaccine [35]. Likewise, a pro-inflammatory gene expression profile within the tumor microenvironment was associated with survival following administration of a protein-based vaccine in patients with metastatic melanoma [36]. Response to CTLA-4 blockade in patients with metastatic melanoma was also correlated with expression of interferon inducible genes and T_H_1 associated markers [37]. Finally, expression of T cell homing genes in the tumor vascular endothelium has also been implicated in mitigating lymphocyte infiltration [38]. Two important implications of these data include the potential for improved patient selection for administration of immunotherapeutics and identifying potential strategies for improved response to immunotherapies in patient populations that would otherwise respond poorly to immunologic interventions.

### T cell intracellular signaling

To understand how the adaptive arm of the immune system is engaged, a basic knowledge of T cell biology and activation can be helpful. T cells detect antigen bound to MHC molecules, with the CD4^+^ T cell subset binding to MHC Class II primarily expressed on APCs while CD8^+^ T cells are activated by binding to MHC Class I, which can be expressed by APCs as well as normal cells. Following initial APC-driven activation, CD8^+^ T cells may later recognize target cells expressing their cognate antigen, resulting in cell-mediated cytotoxicity. For T cells to be fully activated, the APC must provide other signals in addition to the peptide/MHC (signal 1). The B7-CD28 interaction, with B7 expressed on the APC and CD28 on T cells, was one of the first co-stimulatory signaling pathways elucidated [39]. CD28 signaling is complex, but most likely functions in part by increasing T cell expression of anti-apoptotic proteins (e.g. Bcl-xL) and autocrine growth factors like IL-2 [40,41]. Additional co-stimulatory interactions (APC:T-cell) include OX40L-OX40, CD70-CD27, CD137L-CD137, and B7RP1-ICOS [42]. Ultimately, an effective tumor vaccine requires activating APCs to express appropriate co-stimulatory molecules to promote durable anti-tumor immune responses through intracellular signaling cascades.

Following T cell engagement with appropriately activated APCs, intracellular signaling results in activation of three signaling cascades: NF-AT, NF-kB, and AP-1 [43]. Of these, the NF-AT pathway is particularly interesting, since NF-AT signaling in the absence of AP-1 results in immune tolerance, whereas *in vivo* blockade of NF-AT decreases both T cell activation and limits tolerance [44,45]. Recent data showed that the Adenosine 2a Receptor (A2aR) is a component of the negative feedback loop for T cell activation that is upregulated during T cell activation, and blockade of A2aR has been shown to increase the efficacy of tumor vaccines in pre-clinical models [46].

T cell activation is clearly influenced by the spectrum of cytokines present during antigen recognition, and several cytokines exert their immunologic effects by modulating the function of STAT proteins during T cell activation. In that regard, STAT4 has thus far been demonstrated to be crucial to T cell mediated anti-tumor immune responses. IL-12 activates STAT4, which in turn skews T cells toward a T_H_1 phenotype and IFN-γ production [6]. Recent data show that, in addition to the canonical pathways such as NF-AT and AP-1, the mTOR (mammalian target of rapamycin) pathway also plays a critical role in T cell activation and function. mTOR is an evolutionarily conserved serine/threonine protein kinase central to integrating nutrient and hormone signaling pathways [47], which in turn regulates SGK1, a protein important in epithelial survival. In preclinical models, SGK1 knockout mice had increased response to tumor immunotherapy compared to mice with functional SGK1, suggesting that mTOR up-regulation dampens or inhibits anti-tumor immunity (unpublished data from Dr. Jonathan D. Powell, Johns Hopkins University). In summary, T cell activation is relatively complex, and is generally only partially understood, but plays a critical role in generating an adaptive anti-tumor immune response.

### Memory T-cells

Following initial activation, a minority (5-10%) of T cells become long-lived memory cells with enhanced functional responses upon antigen re-encounter as compared to naïve T cells. For cancer immunotherapy the importance of generating functional memory cells is two-fold. First, the presence of memory cells could potentially decrease metastatic spread and prevent tumor re-growth after an initial response. Second, memory cells could limit de novo induction of a second malignancy. The importance of tumor infiltrating memory T cells is further illustrated with the novel *Immunoscore*, which has demonstrated prognostic and predictive value in colorectal cancer through the quantification of tumor infiltrating cytotoxic effector cells and memory T cells [48]. Current understanding of memory T cells is derived largely from the study of the immune response to microbes; however, in the absence of good models of memory induction in tumor bearing animals or humans, one can reasonably extrapolate these findings to anti-tumor responses. It was originally hypothesized that memory T cells were selected randomly from the naïve T cell pool during the expansion phase of an effector response; but it is now thought that some T cells are intrinsically more likely than others to persist after an initial response as memory cells [49]. Thus, cells more likely to become memory cells express the IL-7 Receptor alpha (IL-7Rα) chain, CD27 (Tumor Necrosis Factor Receptor Superfamily 7), BCL-2, and downregulate the effector molecule KLRG1 (Killer Cell Lectin-like Receptor Subfamily G1) [50-52]. However, this expression profile is not exclusive to memory cells, as there are short-lived IL-7Rα^+^ cells that also have high expression of KLRG1 [50]. Memory cells can be divided into three relatively distinct subsets including: (1) “effector-memory” having more cytotoxic function; (2) “central-memory” cells, which likely represent the more classic quiescent memory cell with high proliferative capacity once re-stimulated; and (3) “tissue-resident-memory” associated with an organ-specific distribution *in vivo*[53].

Multiple models exist for T cell diversification and long-term cell fate. The Separate-Precursor model, which is less feasible compared to other models that will be discussed, states that cells are pre-programmed in the thymus for subsequent development into a memory cell or an effector cell [53]. The Decreasing-Potential model postulates that repetitive exposure to antigen and stimuli drives T cells away from a memory phenotype towards terminal effector differentiation [53]. The Signal-Strength model proposes that memory cell development is dependent on the overall strength of the signals received by the T cell through antigen (signal 1), costimulation (signal 2), and pro-inflammatory cytokines (signal 3) [53]. Finally, the Asymmetric-Cell-Fate model posits that when a T cell encounters an APC and divides while still bound to the APC, the daughter cell that remains attached to the DC (immunologic synapse) will receive greater signals through TCR and costimulation resulting in greater potential for terminal effector differentiation. Conversely, the daughter cell that is not adjacent to the immunologic synapse will have greater potential for memory cell differentiation [53].

Differential transcription factor expression is associated with the memory versus effector transcriptome. T-bet, BLIMP1, ID2, and STAT4 activity are associated with effector T cells [53,54]. Similarly, EOMES, BCL-6, and STAT3 activity are more associated with memory T cells [53,55]. Contemporary modeling supports a graded expression of these transcription factors resulting ultimately in the final lymphocyte phenotype. Additionally, there are metabolic differences between memory and effector T cells. Interestingly, memory cells—being more quiescent as compared to effector T cells—sustain ATP through fatty acid oxidation, whereas effector T cells utilize aerobic glycolysis and lipid synthesis [56]. In that regard, mTOR is at least partially responsible for the aerobic metabolism found in effector T cells [57].

When T cells are continually exposed to antigen, as is often the case for lymphocytes specific for tumor-associated antigens, there exists the potential for such T cells to become “exhausted”. T cell exhaustion is characterized by loss of effector cytokine production (IL-2, TNF-α, and IFN-γ), impaired proliferation, and decreased cytotoxicity. Whereas memory T cells require IL-7 and IL-15 for maintenance, exhausted T cells appear to be maintained via continued exposure to antigen. Exhausted T cells also have a distinct transcriptome with upregulation of BLIMP1, EOMES, BATF and down-regulation of T-bet. Furthermore, exhausted lymphocytes express negative regulatory surface molecules including PD-1, LAG-3, TIM-3, 2B4, and CD160 [58]. Exhaustion is clearly reversible in some cases, as PD-1 blockade in a viral model of exhaustion was able to rescue the T cells from their exhausted phenotype [59], and blocking these immune checkpoint molecules associated with exhaustion is showing promise in multiple clinical trials [60]. Taken together, these new insights into memory cell differentiation and function offer multiple novel avenues for intervention in terms of generating a productive anti-tumor response.

### Tumor antigens and immunogenicity

T cells recognize antigen in the form of small peptides, derived from proteolysed substrates, and presented in the context of MHC molecules. MHC molecules are genetically diverse, and for each MHC variant, only specific peptide sequences from a given antigen are able to bind for presentation to T cells and subsequent induction of anti-tumor immune responses. Understanding the specific antigens recognized by the immune system and the specific peptide sequences presented on MHC can be important in improving immunotherapies directed against a specific antigen. Several approaches have been used to identify tumor specific antigens, including molecular cloning; sequencing of antigenic peptides; and computer algorithms, each of which has its relative benefits and deficiencies.

Multiple processing pathways exist for proteolysis of antigen through the proteosome and presentation in MHC molecules [61]. Determinants of a peptide’s ability to induce an immune response (i.e. its “antigenicity”) include its affinity to the MHC, as well as the affinity of the peptide/MHC complex for a given T Cell Receptor (TCR). A critical facet of this interaction is a set of amino acids which are integral to MHC binding, so-called MHC-anchor-residues. To induce more robust immune responses, it is possible to modify antigenic peptides in several ways. MHC variable peptides (MVP), for example, are peptides designed with amino acid point changes involving MHC-contact residues, usually optimized for improved MHC affinity. Conversely, altered peptide ligands (APL) are peptides with amino acid substitutions designed to optimize interactions with the T cell receptor. These altered peptides have been used in an attempt to augment immune response against a specific antigen [62]. MVPs/APLs have been used in both the preclinical and clinical setting resulting in improved immunogenicity for a number of tumor antigens, including gp100, CEA, and NY-ESO/LAGE-1 [63-65]. Understanding the specific antigen/peptide associated with anti-tumor immune responses allows for monitoring of ongoing immunologic responses with *ex vivo* studies including enumeration via tetramer and functionality via IFN-γ production, ELISPOT, lytic activity, functional avidity, and replicative history assayed via enumerating telomere length.

The term “cancer vaccine” encompasses a variety of approaches sharing the common goal of activating and expanding a population of specific T cells to generate an anti-tumor response. A variety of vaccine approaches have been explored, including synthetic peptides, recombinant virus-like particles (VLP), naked/stabilized nucleic acids, recombinant viruses, recombinant bacteria, and dendritic cells. One notable facet of cancer vaccines is that they must provide antigen (signal 1) in addition to a second signal (signal 2) to elicit full effector function. The addition of an appropriate adjuvant to a vaccine (i.e. a “danger” signal [9]), can be important in providing Signal 2. Despite a great deal of work, only two cancer vaccines have been approved for clinical use, including Oncophage (Russia, 2008) and Provenge (sipuleucel-T) (USA, 2010). The PSA-targeting viral vaccine ProstVac VF is currently in phase III trials worldwide [17,66]. Although monitoring vaccine responses in peripheral blood is challenging, recent studies suggest that patients treated with sipuleucel-T do mount detectable antigen-specific T and B cells responses, which correlate to some degree with outcome [67]. Clinically, single agent efficacy of most cancer vaccines is less obvious, with objective clinical responses rarely detected [68]. Although multiple mechanisms may underlie this observation, data showing expression of immune checkpoint molecules like PD-1 and CTLA-4 on tumor-specific lymphocytes suggests that combining immune checkpoint blockade with vaccination might be one way to optimize a vaccine-initiated anti-tumor immune response [69].

### Coinhibition and costimulation in cancer immunotherapy

As discussed above, the T-cell/APC interaction involves engagement of the TCR with the antigen-MHC complex; in addition, costimulation/coinhibition interactions also occur and these secondary receptor/ligand binding events ultimately affect downstream T cell responses. Classically, costimulation involves the interaction of B7-CD28–disruption of this interaction by CTLA-4 expression on T cells, with associated tight binding to B7 is referred to as co-inhibition [39,70]. Early preclinical studies demonstrated that blockade of CTLA-4 could mitigate inhibition of anti-tumor immune responses [71]. This finding was eventually confirmed clinically in Phase III trials in patients with metastatic melanoma [72]. In addition to CTLA-4, tumor infiltrating lymphocytes may express the negative regulatory receptors PD-1, LAG-3, TIM-3 and others [73-75]. Preclinical blockade of these pathways results in improved anti-tumor immunity [76-78]. Interestingly, a single tumor-infiltrating lymphocyte may express multiple immune checkpoint molecules simultaneously, so it is not surprising that combined blockade suggests improved efficacy in preclinical models [77]. The ligand for PD-1 is PD-L1, and expression of PD-L1 in tumors correlated with patient response to anti-PD-1 therapy [79]. These data would suggest that there could be potential biomarkers for checkpoint blockade therapy. Current preclinical studies are combining checkpoint blockade with tyrosine kinase inhibitors, radiation therapy and cancer vaccines.

### Adoptive cellular therapy

Adoptive T cell therapy allows for *ex vivo* stimulation of lymphocytes in a non-tolerizing environment followed by re-infusion of activated T cells into patients. There are varying sources and types of T cells used for adoptive therapy, these include tumor infiltrating lymphocytes (TILs), T cells engineered to express a cancer-specific TCR, and T cells engineered to express a chimeric antigen receptor (CAR) that combines the extracellular portion of an antibody with the T cell receptor signaling machinery. Of these approaches, expanded TILs are the least labor intensive to produce, yet require an invasive procedure to obtain. Additionally, maintenance of TILs after adoptive transfer usually requires high dose IL-2, which results in significant toxicity. Clinical response rates in patients with metastatic melanoma treated with expanded TILs is impressive, approximately 50% in several studies [80]. Furthermore, pretreatment of patients with lymphodepletion can result in a greater proportion of clinical responses and more durable responses [81]. As previously discussed, host T cells can be re-engineered to express CAR in place of the TCR. The CAR expresses an antibody Fv region in place of the extracellular domain of the TCR allowing the T cell to recognize whole antigen as opposed to MHC-restricted antigen [82]. This approach is efficient and results in T cells with uniform specificity, but is limited to some degree by transduction efficiency and potential toxicity. Another approach to adoptive T cell therapy is the use of endogenous tumor-specific T cells. This approach involves pheresis of circulating tumor-specific T cells, *in vitro* expansion and activation, and lastly reintroduction into the host via adoptive transfer [83]. This approach is considered to be the most physiologic, but is most labor intensive as it involves multiple pheresis sessions and significant laboratory labor for the expansion and activa-tion steps. Given the recent high-profile success of chimeric antigen receptor modified T cells for patients with CLL, these approaches are attracting increasing attention and enthusiasm [29,84].

## Conclusions

The immune system is exquisitely poised to recognize and distinguish self from non-self. Further, the immune system is able to recognize self from “altered-self”, which is the case for cancer. Although clinically apparent malignancies have likely circumvented endogenous anti-tumor immune responses, immunotherapy has the potential to augment responses in order to mitigate tumor progression. As reviewed above, immune responses can be divided between innate and adaptive responses. Innate immune responses recognize general patterns of non-self (e.g. double-stranded RNA, single-stranded DNA, LPS) and are able to initiate pro-inflammatory responses which in turn can attract immune components leading to adaptive immunity. Adaptive immunity is defined by acquired immunity to specific antigens, and is mediated through B cells via antibody secretion and T cells through cell-mediated immunity. Dendritic cells are antigen presenting cells which function at the crossroads of innate and adaptive immunity and are able to cross-present antigen to, and activate, T cells. Dendritic cells are a target of various immunotherapeutic approaches either through the use of adjuvant cytokines which activate dendritic cells or more directly through the use of dendritic cell vaccines. Antibodies produced by B cells are highly specific for cognate antigen and have been engineered through various mechanisms to simultaneously target the tumor antigen and potentiate anti-tumor immune responses. T cells are ultimately responsible for cell-mediated immune responses, which are thought to be the most important mechanism of immune related tumor killing for solid malignancies. T cells can be activated through antibody blockade of inhibitory signaling, vaccination, or *ex vivo* stimulation followed by adoptive transfer into patients. A more complete understanding of the cellular and molecular components of the tumor-immune system interaction is crucial to the development of rational and efficacious immunotherapies in the future. This primer serves as a starting point for the cancer biologist and budding immunotherapist to better understand and appreciate the past, present, and future of immunotherapeutics.

## Competing interests

The authors declare that they have no competing interests.

## Authors’ contributions

TH and CG contributed equally to the background research and writing of this review. Both authors read and approved the final manuscript.
